# Supplementary material for: Consistency of feature attribution in deep learning architectures for multi-omics
Source: Sci Rep. 2026 Aug 18;16:25890. doi: 10.1038/s41598-026-58312-5 (PMC13486497; doi:10.1038/s41598-026-58312-5)
Supplement: Supplementary file 1 [file 41598_2026_58312_MOESM15_ESM.docx]

For each of the 27 runs (15 mean and 12 concatenation) of the performance of features analysis on the binary classification viral dataset, SHAP values for each protein were summed and used to order proteins in a set from highest to lowest summed SHAP value. Gene Ontology (GO) [1] was extracted from the molecular signatures database [2] with the R package *msigdbr* [3]. 826 of the 2146 proteins had associated GO information. Set enrichment was conducted with *fgsea* [4] with no limitations to set size, and p-values were reported for each set. The top 10 significant sets by p-value (lowest p-value) were then plotted in a presence/absence heat map. The x-axis corresponds to model run, the y-axis corresponds to GO term, and the color represents whether the given term was in the top ten functions ranked by gene set enrichment.

If the models were consistently ranking different biomolecules all corresponding to the same functional terms, this would be an indication that while there is feature ranking instability, the instability is a product of flexible "swapping" amongst biomolecules associated with the same GO terms. Because we see that the GO terms themselves are different across runs, we have higher confidence that the variation in SHAP rankings is tied to an underlying variation in GO term / biological function rankings. This ranking instability in the GO terms supports the hypothesis that conclusions drawn from SHAP importance scores may be unreliable.

 Figure S1: Presence/Absence heat map comparing top ranked GO terms (y-axis) across experimental runs (x-axis). Blue hexes correspond to GO term being in the top 10 ranked GO terms determined with gene set enrichment analysis.

[1]	The Gene Ontology Consortium *et al.*, “The Gene Ontology knowledgebase in 2023,” *Genetics*, vol. 224, no. 1, p. iyad031, May 2023, doi: 10.1093/genetics/iyad031.

[2]	A. Liberzon, C. Birger, H. Thorvaldsdóttir, M. Ghandi, J. P. Mesirov, and P. Tamayo, “The Molecular Signatures Database Hallmark Gene Set Collection,” *Cell Syst.*, vol. 1, no. 6, pp. 417–425, Dec. 2015, doi: 10.1016/j.cels.2015.12.004.

[3]	I. Dolgalev, *msigdbr: MSigDB Gene Sets for Multiple Organisms in a Tidy Data Format*. (Jul. 21, 2025). Accessed: Jan. 16, 2026. [Online]. Available: https://cran.r-project.org/web/packages/msigdbr/index.html

[4]	G. Korotkevich, V. Sukhov, N. Budin, B. Shpak, M. N. Artyomov, and A. Sergushichev, “Fast gene set enrichment analysis,” Feb. 01, 2021, *bioRxiv*. doi: 10.1101/060012.
