# Supplementary material for: Consistency of feature attribution in deep learning architectures for multi-omics
Source: Sci Rep. 2026 Aug 18;16:25890. doi: 10.1038/s41598-026-58312-5 (PMC13486497; doi:10.1038/s41598-026-58312-5)
Supplement: Supplementary file 2 [file 41598_2026_58312_MOESM2_ESM.docx]

Layer Sizes and Noise Levels for Added-Noise Ablation Experiments

We specify the data types used, the layer sizes for the first and second network formatted as “[[first network sizes], [second network sizes]]” as well as the number of noise features appended to the input of the first marginal network.

**Configuration 1**

Metabolites, Proteins

| layer_sizes | n_noise |
| --- | --- |
| [[64, 64], [512, 512]] | 100 |
| [[64, 64], [512, 512]] | 500 |
| [[64, 64], [512, 512]] | 1000 |
| [[64, 64], [512, 512]] | 2500 |
| [[64, 64], [512, 512]] | 4000 |
| [[73, 73], [439, 439]] | 100 |
| [[95, 95], [339, 339]] | 500 |
| [[111, 111], [292, 292]] | 1000 |
| [[137, 137], [236, 236]] | 2500 |
| [[154, 154], [211, 211]] | 4000 |

**Configuration 2**

Metabolites, Lipids

| layer_sizes | n_noise |
| --- | --- |
| [[128, 128], [256, 256]] | 100 |
| [[128, 128], [256, 256]] | 500 |
| [[128, 128], [256, 256]] | 1000 |
| [[128, 128], [256, 256]] | 2500 |
| [[128, 128], [256, 256]] | 4000 |
| [[148, 148], [219, 219]] | 100 |
| [[192, 192], [169, 169]] | 500 |
| [[223, 223], [145, 145]] | 1000 |
| [[276, 276], [117, 117]] | 2500 |
| [[309, 309], [105, 105]] | 4000 |

**Configuration 3**

Metabolites, Proteins

| layer_sizes | n_noise |
| --- | --- |
| [[64, 64], [128, 128]] | 100 |
| [[64, 64], [128, 128]] | 500 |
| [[64, 64], [128, 128]] | 1000 |
| [[64, 64], [128, 128]] | 2500 |
| [[64, 64], [128, 128]] | 4000 |
| [[86, 86], [94, 94]] | 100 |
| [[145, 145], [56, 56]] | 500 |
| [[195, 195], [41, 41]] | 1000 |
| [[299, 299], [27, 27]] | 2500 |
| [[375, 375], [21, 21]] | 4000 |

**Configuration 4**

Metabolites, Lipids, Proteins

| layer_sizes | n_noise |
| --- | --- |
| [[128, 128], [256, 256], [512, 512]] | 100 |
| [[128, 128], [256, 256], [512, 512]] | 500 |
| [[128, 128], [256, 256], [512, 512]] | 1000 |
| [[128, 128], [256, 256], [512, 512]] | 2500 |
| [[128, 128], [256, 256], [512, 512]] | 4000 |
| [[148, 148], [219, 219], [438, 438]] | 100 |
| [[192, 192], [169, 169], [338, 338]] | 500 |
| [[223, 223], [145, 145], [290, 290]] | 1000 |
| [[276, 276], [117, 117], [234, 234]] | 2500 |
| [[309, 309], [105, 105], [210, 210]] | 4000 |
