# Supplementary material for: Consistency of feature attribution in deep learning architectures for multi-omics
Source: Sci Rep. 2026 Aug 18;16:25890. doi: 10.1038/s41598-026-58312-5 (PMC13486497; doi:10.1038/s41598-026-58312-5)
Supplement: Supplementary file 3 [file 41598_2026_58312_MOESM3_ESM.docx]

This pseudocode details the method used to dynamically adjust the size of the hidden layers when synthetic noise features are injected. The goal is to scale the hidden layer size such that the number of parameters associated with that layer remains approximately consistent or scales inversely with the square root of the increased input dimensionality, thereby maintaining a comparable model complexity despite varying input feature counts.

This specific example demonstrates the calculation for the ICL104 dataset (binary classification, config 1 in supplementary item 2, `**noise-sweep-tables.docx**`), and the calculation is performed analogously for the ICL102 dataset.

```
# parameters
n_noise = 100 # number of noise features
p0_size = 120 # original metabolite size for ICL104
n_params_0 = 64^2 # number of parameters in metabolite base model
n_params_1 = 512^2 # number of parameters in protein base model

# compute multiplier: ratio of noise to size of metabolites
mult = sqrt(1 + (n_noise / p0_size))

# compute new layer size for metabolites (increase layer size)
x_0 = (-1 + sqrt(1 + 4 * mult * n_params_0)) / 2

# compute new layer size for proteomics (decrease layer size)
x_1 = (-1 + sqrt(1 + 4 * n_params_1 / mult)) / 2
```
