# Supplementary material for: Consistency of feature attribution in deep learning architectures for multi-omics
Source: Sci Rep. 2026 Aug 18;16:25890. doi: 10.1038/s41598-026-58312-5 (PMC13486497; doi:10.1038/s41598-026-58312-5)
Supplement: Supplementary file 4 [file 41598_2026_58312_MOESM4_ESM.docx]

To compare our simplified, non-variational model against Lee and van der Schaar’s model, we retrieved the TCGA data from the Broad Institute firehose (<https://gdac.broadinstitute.org/>). Following their work, we reduced the feature dimension of each of the 4 views to 100 features using kernel-PCA with a polynomial kernel, and labels were created by assigning 0 to all individuals who had greater than a 1-year survival rate and 1 otherwise (non-mortality censored samples were dropped). Data was split into 70/15/15 train/validation/test sets, where the validation set was used to select hyperparameters of the loss function, learning rate, and number of epochs.

During training, we set the alpha constants in the class-balanced focal loss to be the inverse of the frequency of the positive and negative classes (0.9391 and 0.1609 respectively), and the gamma constant to be 3. To deal with missing views, when we average outputs of each marginal model, we simply only consider the outputs of the models with non-missing views. All 4 views were included during training, including view-missing samples, which corresponds to Lee and van der Schaar’s (4-view, incomplete) scenario. The model was trained for 40 epochs with a learning rate of 0.0005 and achieved 0.81 AUROC on the test set, a marginal improvement over their model, which achieved 0.801 AUROC. Though our data splits differ from theirs slightly, this result suggests our model is a reasonable competitor to theirs in this particular task.
