# Supplementary material for: Consistency of feature attribution in deep learning architectures for multi-omics
Source: Sci Rep. 2026 Aug 18;16:25890. doi: 10.1038/s41598-026-58312-5 (PMC13486497; doi:10.1038/s41598-026-58312-5)
Supplement: Supplementary file 5 [file 41598_2026_58312_MOESM6_ESM.docx]

## Item numbers referenced in the main document, their file names, and brief descriptions.

1. **`architecture-sweep-tables.docx`**: Four tables of layer sizes for the marginal models and combination layer as well as combination scheme and dataset used.
2. **`noise-sweep-tables.docx`**: Four tables of layer sizes and added noise levels for the experiments on robustness of SHAP values.
3. **`binary-classification-clustering-performance-configs-1-2.csv`**: Full results table containing (among many other values) the classification and performance metrics for the first and second configuration groups from supplementary item 1.
4. **`multiclass-classification-clustering-performance-configs-3-4.csv`**: Full results table containing (among many other values) the classification and performance metrics for the third and fourth configuration groups from item 1.
5. **`noise-exp-results-config-{i}.csv`:** (4 files, with i = 1,2,3,4) Each file contains full results from the configuration specified by the i^th^ item in supplementary item 2.
6. **`tau-v-noise-128-256-metab-lip-shap-deepimv.png`**: Image of degradation of shap value correlation as noise increases for the DeepIMV model of Lee and van der Schaar.
7. **`tau_v_bottom_ranks_static_metab_v_pro_standard.png`**: Violin plots of the SHAP values of the top (i.e. ranks 1,2,3) and bottom ranked biomolecules. This differs from Figure 3 in the paper, in that we only compute rank change among the metabolites, and do not observe the drastic change in ranks as noise increases.
8. **`TCGA-comparison-details.docx`**: Description of modeling efforts to compare our simplified, non-variational model against Lee and van der Schaar’s model.
9. **`dynamic-layer-computation-pseudocode.docx`**: Pseudocode to calculate the dynamic model layer sizes reported in **noise-sweep-tables.docx**.
10. **`classification-AUC-wilcoxon.csv`**: Mean AUC, standard deviation of the AUC, and Wilcoxon signed rank test results for the binary and multiclass classification experiments.
11. **`clustering-metrics-summarized.csv`**: Average V-measure and Adjusted Rand Index computed on agglomerative and kmeans clustering results for the binary and multiclass classification experiments.
12. **`GO-enrichment.docx`**: Enrichment analysis to explore feature instability of GO terms.
