# Supplementary material for: Consistency of feature attribution in deep learning architectures for multi-omics
Source: Sci Rep. 2026 Aug 18;16:25890. doi: 10.1038/s41598-026-58312-5 (PMC13486497; doi:10.1038/s41598-026-58312-5)
Supplement: Supplementary file 6 [file 41598_2026_58312_MOESM7_ESM.docx]

Layer Sizes and Combination Schemes for Comparing Classification and Clustering Performance of Different Architectures

Parameter key:

- hidden_sizes: the sizes of the hidden layers of each network (marginal model) for the metabolites, lipids, proteins, in the format [[metabolites layer sizes], [lipid layer sizes], [protein layer sizes]].
- combine_fn: Whether the final hidden representations of the marginal models are combined via concatenation or averaging.
- hidden_dim: The size of the layer after the combination step and before the final prediction layer.

All parameters are swept over in combination, for example, in Configuration 1, all settings of hidden size are tested with hidden_dim=64, as well as hiden_dim=32, resulting in 12 total runs.

**Configuration 1:**

| Parameter | Values |
| --- | --- |
| hidden_sizes | [[92, 64], [152, 64], [1105, 64]]  [[76, 32], [136, 32], [1089, 32]]  [[68, 16], [128, 16], [1081, 16]]  [[92, 64], [184, 128], [1645, 1144]]  [[76, 32], [152, 64], [1359, 572]]  [[68, 16], [136, 32], [1216, 286]] |
| combine_fn | concat |
| hidden_dim | 64, 32 |
| dataset | ICL-104 (binary) |

**Configuration 2:**

| Parameter | Values |
| --- | --- |
| hidden_sizes | [[128], [128], [128]]  [[92, 64], [152, 64], [1105, 64]]  [[76, 32], [136, 32], [1089, 32]]  [[68, 16], [128, 16], [1081, 16]]  [[64, 8], [124, 8], [1077, 8]] |
| combine_fn | mean |
| hidden_dim | 64, 32, 16 |
| dataset | ICL-104 (binary) |

**Configuration 3:**

| Parameter | Values |
| --- | --- |
| hidden_sizes | [[42, 8], [130, 8], [1265, 8]]  [[46, 16], [134, 16], [1269, 16]]  [[54, 32], [142, 32], [1277, 32]]  [[70, 64], [158, 64], [1293, 64]]  [[128], [128], [128]] |
| combine_fn | mean |
| hidden_dim | 64, 32, 16 |
| dataset | ICL-102 (multiclass) |

**Configuration 4:**

| Parameter | Values |
| --- | --- |
| hidden_sizes | [[46, 16], [134, 16], [1269, 16]]  [[54, 32], [142, 32], [1277, 32]]  [[70, 64], [158, 64], [1293, 64]]  [[40, 4], [132, 13], [1327, 131]]  [[42, 8], [139, 26], [1392, 262]]  [[46, 16], [152, 52], [1523, 524]]  [[54, 32], [178, 104], [1785, 1048]]  [[70, 64], [230, 209], [2310, 2097]] |
| combine_fn | concat |
| hidden_dim | 64, 32 |
| dataset | ICL-102 (multiclass) |
